# Comparison of Aminoglycoside Antibiotics and Cobalt Chloride for Ablation of the Lateral Line System in Giant Danios

**DOI:** 10.1093/iob/obac012

**Published:** 2022-03-21

**Authors:** P J Mekdara, S Tirmizi, M A B Schwalbe, E D Tytell

**Affiliations:** Department of Biology, Tufts University, 200 Boston Avenue, Ste 4700, Medford, MA 02155, USA; National Institute of Neurological Disorders and Stroke, National Institutes of Health, 35 Convent Drive, Building 35, 2B-1004, Bethesda, MD 20892, USA; Department of Biology, Tufts University, 200 Boston Avenue, Ste 4700, Medford, MA 02155, USA; Department of Biology, Tufts University, 200 Boston Avenue, Ste 4700, Medford, MA 02155, USA; Department of Biology, Lake Forest College, 555 N Sheridan Road, Lake Forest, IL 60045, USA; Department of Biology, Tufts University, 200 Boston Avenue, Ste 4700, Medford, MA 02155, USA

## Abstract

The mechanoreceptive lateral line system in fish is composed of neuromasts containing hair cells, which can be temporarily ablated by aminoglycoside antibiotics and heavy metal ions. These chemicals have been used for some time in studies exploring the functional role of the lateral line system in many fish species. However, little information on the relative effectiveness and rate of action of these chemicals for ablation is available. In particular, aminoglycoside antibiotics are thought to affect canal neuromasts, which sit in bony or trunk canals, differently from superficial neuromasts, which sit directly on the skin. This assumed ablation pattern has not been fully quantified for commonly used lateral line ablation agents. This study provides a detailed characterization of the effects of two aminoglycoside antibiotics, streptomycin sulfate and neomycin sulfate, and a heavy metal salt, cobalt (II) chloride hexahydrate (CoCl_2_), on the ablation of hair cells in canal and superficial neuromasts in the giant danio (*Devario aequipinnatus*) lateral line system, as a model for adult teleost fishes. We also quantified the regeneration of hair cells after ablation using CoCl_2_ and gentamycin sulfate to verify the time course to full recovery, and whether the ablation method affects the recovery time. Using a fluorescence stain, 4-Di-2-ASP, we verified the effectiveness of each chemical by counting the number of fluorescing canal and superficial neuromasts present throughout the time course of ablation and regeneration of hair cells. We found that streptomycin and neomycin were comparably effective at ablating all neuromasts in less than 12 h using a 250 μM dosage and in less than 8 h using a 500 μM dosage. The 500 μM dosage of either streptomycin or neomycin can ablate hair cells in superficial neuromasts within 2–4 h, while leaving those in canal neuromasts mostly intact. CoCl_2_ (0.1 mM) worked the fastest, ablating all of the hair cells in less than 6 h. Complete regeneration of the neuromasts in the lateral line system took 7 days regardless of chemicals used to ablate the hair cells. This study adds to the growing knowledge in hearing research about how effective specific chemicals are at ablating hair cells in the acoustic system of vertebrates.

## Resumen

El sistema de la línea lateral mecanorreceptora en los peces está compuesto de neuromastos que contienen células ciliadas, que pueden ser ablacionadas temporalmente con antibióticos aminoglucósidos e iones de metales pesados. Estos productos químicos se han utilizado durante algún tiempo en estudios que exploran el papel funcional del sistema de línea lateral en muchas especies de peces. Sin embargo, se dispone de poca información sobre la eficacia relativa y la tasa de acción de estos productos químicos para la ablación. En particular, se cree que los antibióticos aminoglucósidos afectan a los neuromastos de canal, que están posicionados en los canales de hueso o del tronco, de manera diferente a los neuromastos superficiales, que están posicionados directamente sobre la piel. Este supuesto patrón de ablación no se ha cuantificado por completo para los agentes de ablación de la línea lateral de uso común. Este estudio proporciona una caracterización detallada de los efectos de dos antibióticos aminoglucósidos, sulfato de estreptomicina y sulfato de neomicina, y una sal de metal pesado, hexahidrato de cloruro de cobalto (II) (CoCl_2_), en la ablación de las células ciliadas en neuromastos superficiales y de canal en el sistema de línea lateral en el danio gigante (*Devario aequipinnatus*), como un modelo para peces teleósteos adultos. También cuantificamos la regeneración de las células ciliadas después de la ablación con CoCl_2_ y sulfato de gentamicina para verificar el curso del tiempo hasta la recuperación completa y si el método de ablación afecta el tiempo de recuperación. Usando una tinción de fluorescencia, 4-Di-2-ASP, verificamos la efectividad de cada químico contando el número de fluorescentes neuromastos superficialises y de canal presentes a lo largo del tiempo de ablación y regeneración de las células ciliadas. Encontramos que la estreptomicina y la neomicina fueron comparablemente efectivas en la ablación de todos los neuromastos en menos de 12 horas usando una dosis de 250 μM y en menos de 8 horas usando una dosis de 500 μM. La dosis de 500 μM de estreptomicina o neomicina puede eliminar las células ciliadas en los neuromastos superficiales dentro de 2 a 4 horas, dejando las celulas de los neuromastos de canal casi intactas. CoCl_2_ (0,1 mM) funcionó más rápido, eliminando todas las células ciliadas en menos de 6 horas. La regeneración completa de los neuromastos en el sistema de la línea lateral tomó siete días, independientemente de los productos químicos utilizados para extirpar las células ciliadas. Este estudio se suma al creciente conocimiento en la investigación auditiva sobre cuán efectivos son los químicos específicos para eliminar las células ciliadas en el sistema acústico de los vertebrados.

## Introduction

Fish sense fluid movement immediately around their body with their mechanosensory lateral line system, relying on this sensory system to navigate their environment and to detect conspecifics, prey, and predators. This system is comprised of two types of receptors called neuromasts, which contain clumps of hair cells similar to those found in the inner ear of birds and mammals. Researchers often use chemical or surgical techniques to ablate, or to remove the function of, the hair cells in the lateral line system to identify and compare its role in behaviors such as schooling (Partridge and Pitcher 1980; Mekdara et al. 2018), feeding (Carrillo and McHenry 2016; Schwalbe et al. 2016), escape behaviors (Stewart et al. 2013; Nair et al. 2017), and locomotion (Liao 2006; Voesenek et al. 2018). Specifically, lateral line hair cells are susceptible to damage by ototoxic chemicals, which induce cell death (Monroe et al. 2015). These chemicals include aminoglycoside antibiotics (Song et al. 1995; Harris et al. 2003; Murakami et al. 2003; Owens et al. 2009; Van Trump et al. 2010) and heavy metal ions (Faucher et al. 2006; Olivari et al. 2008; Butler and Maruska 2016). These chemicals are cost-effective, easy to use, and less invasive than surgical methods, which result in permanent loss of lateral line function. Moreover, unlike mammals, fish can rapidly regenerate, or regain the function of, these hair cells, which make fish an ideal animal system to study hair cell ablation and regeneration (e.g., Hernández et al. 2007; Pinto-Teixeira et al. 2015). Here, we characterize the time course of hair cell ablation and hair cell regeneration in neuromasts for several types of commonly used chemicals, as a way to optimize their use for studying the role of the lateral line system in various behaviors. We also tested whether some chemicals may selectively affect hair cells in the two types of neuromasts, which may allow researchers to evaluate their functions separately. For simplicity, we refer to the ablation of a neuromast to indicate the ablation or loss of function of all of the hair cells in the neuromast.

The lateral line system consists of two types of neuromasts, superficial and canal, which have different morphologies and receptive properties (for review see Coombs et al. 2014). Briefly, superficial neuromasts are on the skin surface, while canal neuromasts are in bony canals below the skin or in lateral line scales along the trunk (Kalmijn 1988; Coombs et al. 2014; Webb and Ramsay 2017). It is important to distinguish these two types of neuromasts because they have different sensory functions. Superficial neuromasts are smaller and contain fewer hair cells and are more receptive to low-frequency signals (<50 Hz), while canal neuromasts are larger and contain more hair cells and are more receptive to high-frequency signals (>200 Hz) (Kroese and Schellart 1987; van Netten and Kroese 1987; Mogdans and Bleckmann 2012; Coombs et al. 2014). Collectively, this indicates that superficial neuromasts primarily sense flow velocity, while canal neuromasts primarily sense flow acceleration (Kroese and Schellart 1987; van Netten and Kroese 1987; Denton and Gray 1988; Kalmijn 1988).

Some studies have permanently ablated the lateral line system by surgically cutting the lateral line nerve (e.g., Partridge and Pitcher 1980), while others have reversibly ablated all of the neuromasts in the system by immersing fish in a less invasive aminoglycoside antibiotic or heavy metal ion treatment. Since high doses or long exposures of these chemicals can be toxic to fish, it is useful to screen such chemicals and quantify the effectiveness and ablation time course of tolerable dosages. In addition, some aminoglycoside antibiotics have been thought to selectively ablate canal or superficial neuromasts (Song et al. 1995), although more recent studies have indicated that these chemicals ablate both neuromast types with nearly equal effectiveness (Van Trump et al. 2010).

Recent studies have begun to identify mechanisms behind the chemical ablation of lateral line hair cells. Aminoglycoside antibiotics ablate hair cells in a manner that involves known apoptotic cell death pathways, free radical formations, and blocking of signal transmission through mechanotransduction channels (Owens et al. 2009). These chemicals seem to be taken up by a hair cell through the mechanotransduction channels, because blocking these channels with amiloride or FM1-43 prevents hair cell loss in zebrafish (Owens et al. 2009; Monroe et al. 2015). Once in the cell, the chemicals trigger apoptotic pathways, indicated by the presence of cell death proteases and caspases after exposure to gentamycin and neomycin (Owens et al. 2009). Moreover, inhibiting caspase reduces hair cell death. Cobalt chloride (CoCl_2_) triggers similar apoptotic pathways in hair cells by competitively inhibiting calcium from entering hair cells through voltage-gated channels of the cell membrane (Karlsen and Sand 1987; Stewart et al. 2017). However, it also acts as a nonspecific calcium channel antagonist and could potentially disrupt other sensory systems such as chemosensory systems during prolonged periods of exposure or in high concentrations (Janssen 2000; Butler et al. 2016; Stewart et al. 2017). Long exposures of CoCl_2_ at high concentrations could have prolonged toxic effects even after removal from the solution (Janssen 2000; Butler et al. 2016). Even at low doses (e.g., 0.1 mM), CoCl_2_ might disrupt other sensory systems such as olfactory tissues and chemosensory hair cells, as reported by Butler et al. (2016) in a few teleosts.

In this study, we investigated how four different ototoxic chemicals, streptomycin, neomycin, and gentamycin (all common aminoglycoside antibiotics) and CoCl_2_ (a heavy metal ion in solution) affected hair cells of the giant danio (*Devario aequipinnatus*) lateral line system. Our goal was to optimize the dosage of these chemicals to ablate the lateral line system efficiently for behavioral studies. Giant danios are an ideal animal system to examine the effects of ototoxic chemicals on the lateral line system more generally because they are closely related to zebrafish (*Danio rerio*, Biga and Goetz 2006), are larger than zebrafish, and have greater numbers of superficial neuromasts within clusters on the body. Like zebrafish, giant danios also have neuromasts that are easily visible under a fluorescent microscope, and their lateral line system has been previously characterized (see Mekdara et al. 2018). While many studies have examined the effects of these chemicals on larvae and adult zebrafish (Harris et al. 2003; Murakami et al. 2003; Santos et al. 2006; Owens et al. 2009; Van Trump et al. 2010; Stengel et al. 2017), we expand on previous studies by establishing a standard dose-response curve of superficial and canal neuromasts ablation and regeneration. Thus, our work helps future investigators to select the most appropriate chemical and dosage. Here, giant danios were treated with streptomycin, neomycin, CoCl_2_, or a sham treatment to assess the time course of hair cell ablation, while other individuals were treated with gentamycin, CoCl_2_, or a sham treatment to assess the time course of hair cell regeneration. For all treated fish, hair cells in neuromasts were visualized using a vital fluorescent dye and counted at specific time points. We demonstrated that CoCl_2_ ablates both types of neuromasts faster than streptomycin and neomycin, but both aminoglycoside antibiotics ablated superficial neuromasts more rapidly than canal neuromasts at a higher dosage. After treating fish with either an aminoglycoside antibiotic or a heavy metal ion, hair cells regenerated at the same rate, and the number of neuromasts returned to normal numbers between 3 and 7 days after treatment.

## Materials and methods

### Animals

**Fig. 1:**
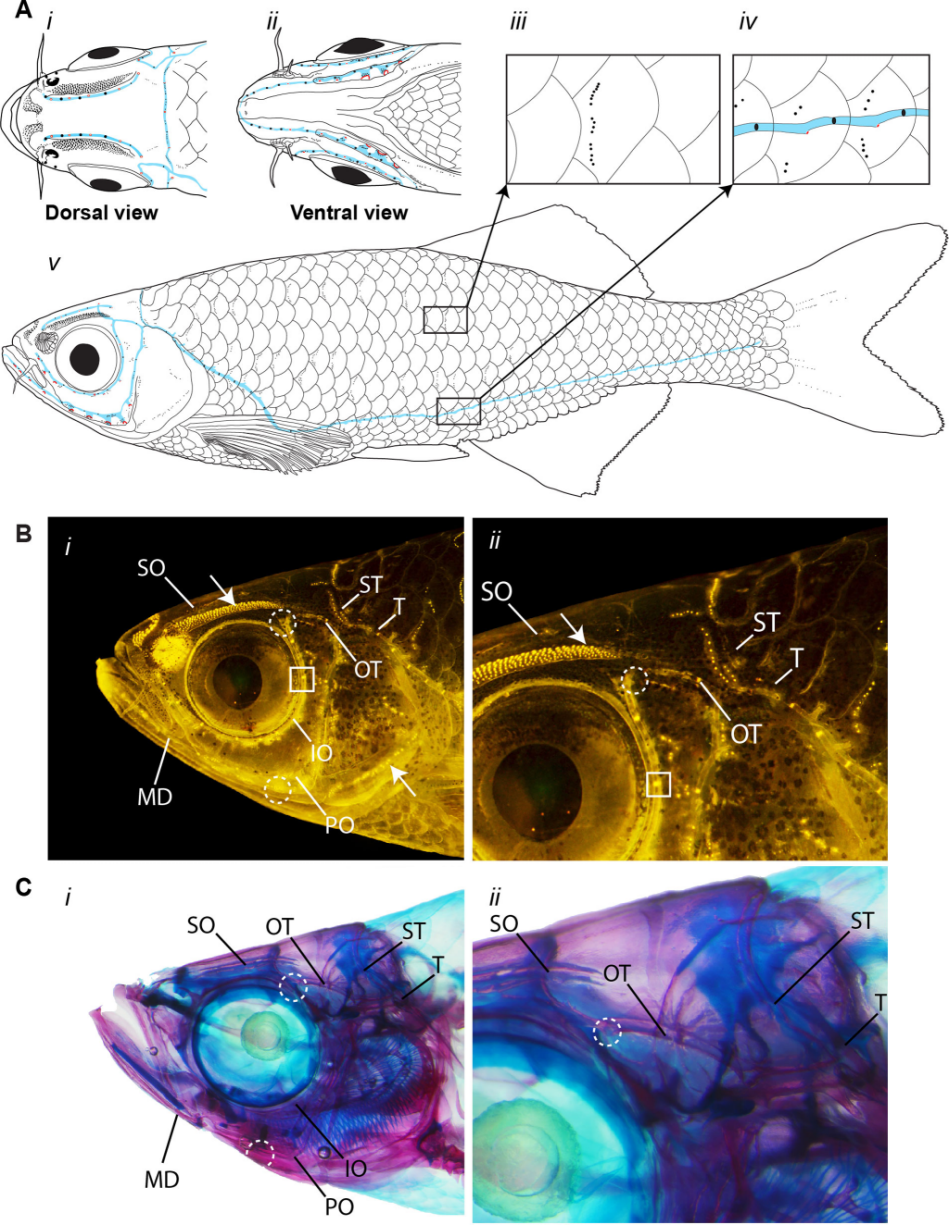
Morphology of the mechanosensory lateral line system and distribution of canal and superficial neuromasts in *D. aequipinnatus.* (**A**) Schematic diagram of the lateral line system of an untreated adult giant danio with (i) a dorsal view of the head, (ii) a ventral view of the lower jaw, (iii) a magnified view of a trunk scale with a vertical stitch of superficial neuromasts, (iv) a magnified view of lateral line scales that contain canal and superficial neuromasts along the trunk canal, and (v) a complete lateral view with canal and superficial neuromasts located on the body. Canal neuromasts (black ovals) are located inside canals (blue shading) and canal pores (red circles) are situated along the canal between each canal neuromasts. (**B**) Lateral view of the head of a giant danio stained with 4-Di-2-ASP showing metabolically active neuromasts as bright yellow dots. Examples of large canal neuromasts are boxed in white, canal pores are circled (dotted) in white, and patches of smaller superficial neuromasts are indicated with white arrows. (**C**) Canal structures and pores can be seen in a cleared and stained fish. SO, supraorbital canal; IO, infraorbital canal; MD, mandibular canal; PO, preopercular canal; OT, optic canal; ST, supratemporal canal; and T, temporal canal, which can extend laterally from head to tail along the full length of the trunk.

We obtained giant danios (*D. aequipinnatus*, Fig. 1) from a commercial supplier (LiveAquaria, Rhinelander, WI, USA). We used three fish per measured time point for each chemical treatment (*n* = 108 fish total). Fish were housed in groups of 25 fish per 40 L aquarium tank under standard conditions under 12:12 dark:light cycles and fed standard goldfish flakes (TetraFin, Blacksburg, VA, USA). All experiments followed an approved Tufts University IACUC protocol (M2015-149 and M2018-103).

### Fluorescent labeling

Functional hair cells in neuromasts positively fluorescence when treated with a vital fluorescent dye (e.g., 4-Di-2-ASP, DASPEI, FM1-43, etc.), while nonfunctional hair cells in neuromasts do not fluorescence (Van Trump et al. 2010; Schwalbe et al. 2016; Mekdara et al. 2018). To assess the time course of hair cell ablation and regeneration of the different treatments, treated fish at their specific time point were placed in a bath of 63 mM 4-Di-2-ASP [4- (4-dimethylamino styryl)-1-methylpyridinium iodide], Sigma-Aldrich, Natick, MA, USA] in standard tank water for 5 min and gently transferred into a bath of buffered 0.02% tricaine methanesulfonate (MS-222, pH = 7.4; Sigma-Aldrich, St. Louis, MO, USA) to anesthetize for imaging. Some fish were euthanized with an overdose of buffered MS-222 in order to take high-quality images. Hair cells in superficial and canal neuromasts were imaged under a dissecting microscope (Leica M165-FC, Leica Microsystems, Wetzlar, Germany) with a green fluorescent protein blue emission filter (wavelength = 425/60 nm excitation range). All images were taken with a DSLR camera (Nikon D5500, Nikon Corporation, Tokyo, Japan) and a 2 s exposure time. Micrographic images were stitched and blended together (using Adobe Photoshop CC, Ottawa, Canada) for a complete view of selected fish.

### Ablation chemical treatments

We treated giant danios with one of three chemicals and at one or two concentrations in standard tank water: 250 or 500 μM streptomycin sulfate (Gold Biotechnology Inc., St. Louis, MO, USA), 250 or 500 μM neomycin sulfate (TekNova, Hollister, CA, USA), or 0.1 mM CoCl_2_ hexahydrate (Sigma-Aldrich, St. Louis, MO, USA). We selected these chemicals and at these concentrations because they have been used in previous studies (e.g., Montgomery et al. 1997; Harris et al. 2003; Murakami et al. 2003; Owens et al. 2009; Buck et al. 2012) but not consistently used with the same species or life stage. Further, we used an established concentration of 0.1 mM for CoCl_2_ because previous work has already identified the overall toxicity of this chemical (Butler et al. 2016; Schwalbe et al. 2016). Fish were placed in these treatments until all hair cells in the lateral line system were completely ablated (≤14 h). We compared these treated fish with additional fish placed in a sham treatment (standard tank water) for 14 h. All fish were handled to the same degree as they were gently moved to and from their respective 10 L treatment tanks. We examined the lateral line system of these fish using fluorescent methods (see below) at specific time points, immediately before the treatment (= 0 h) and at 2-h increments (2–14 h), across each of the chemical treatments. For each time point and treatment, we counted and recorded the number of canal and superficial neuromasts with visible fluorescence until they were no longer visible. For fish in sham treatments, the fluorescent methods confirmed that neither handling nor standard tank water damaged the lateral line system.

### Regeneration chemical treatments

We treated giant danios with 20 μM gentamycin sulfate (Sigma-Aldrich, St. Louis, MO, USA) in standard tank water for 24 h or 0.1 mM CoCl_2_ in standard tank water for 4 h. The number of fluorescing superficial and canal neuromasts was counted and recorded at 1, 2, 3, and 7 days after treatment. We used gentamycin because its effective dosages and exposure times are well documented (Forge and Schacht 2000; Van Trump et al. 2010; Mekdara et al. 2018), while the time course of neuromast recovery using this chemical is less established (but see Pisano et al. 2014). The mechanism for how aminoglycoside antibiotics damage hair cells is thought to be conserved among this class of antibiotics (see the ‘‘Discussion’’ section), and thus only one type of antibiotic was used here.

### Counting neuromasts

**Fig. 2:**
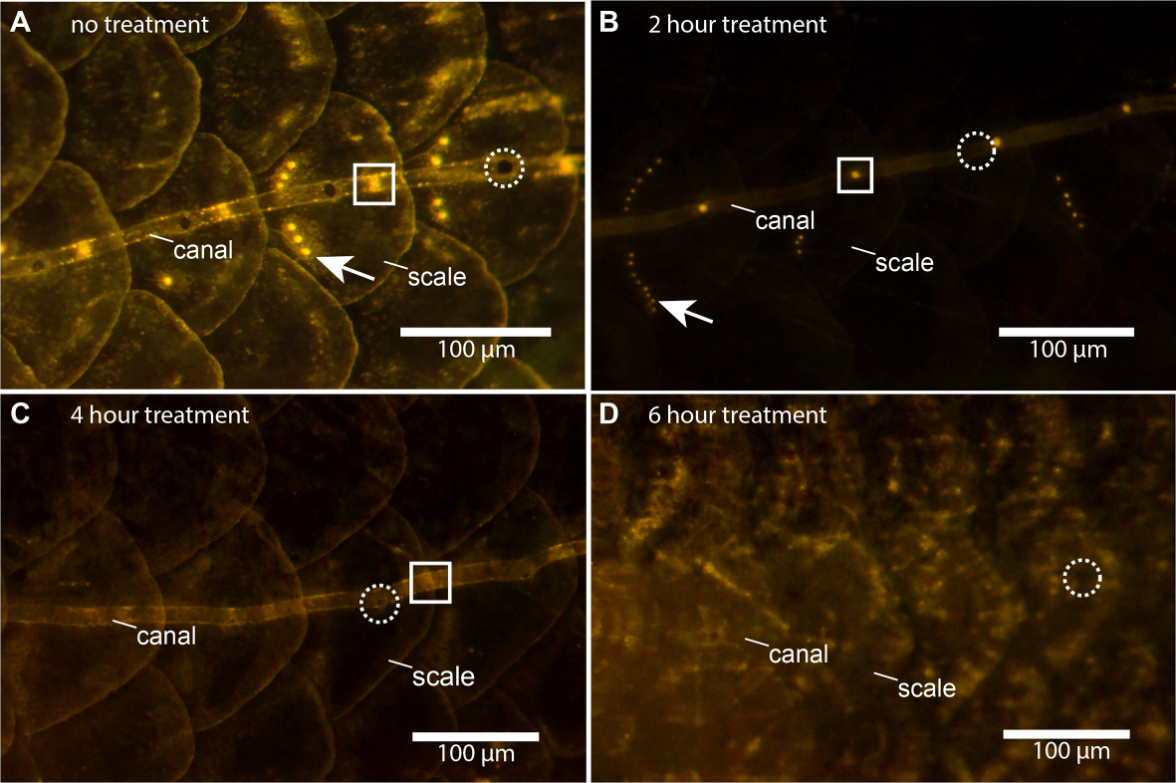
Fluorescent images of canal and superficial neuromasts during 0.1 mM CoCl_2_ ablation treatments in adult giant danios. Bright yellow dots indicate functional hair cells within neuromasts positively stained with a vital fluorescent dye (4-Di-2-ASP), while the lack of yellow dots indicates negative staining and thus successful ablation of the hair cells within a neuromast. (**A**) Normal fluorescence of superficial and canal neuromasts along the trunk canal in an untreated fish. Superficial (white arrow) and canal (white box) neuromasts are illuminated by the dye and show active hair cells. Dashed white circles show a canal pore in between two canal neuromasts. (**B**) Partial fluorescence of superficial and canal neuromasts after treatment with 0.1 mM CoCl_2_ for 2 h. (**C**) No fluorescence of superficial neuromasts and low fluorescence of canal neuromasts (white box), respectively, after treatment with 0.1 mM CoCl_2_ for 4 h. Because canal neuromasts showed very low fluorescence at this time point, they were recorded as functional. Superficial neuromasts are not visible and were marked as completely ablated. (**D**) No fluorescence of superficial and canal neuromasts after treatment with 0.1 mM CoCl_2_ for 6 h. A canal pore (dashed white circle) remains visible despite the loss of fluorescence in neuromasts. All images are at 120× magnification.

We define “ablation” as the complete lack of fluorescence in the hair cells of a neuromast treated with 4-Di-2-ASP (<10%), indicating that all the hair cells are nonfunctional. All visible superficial and canal neuromasts on one side of each fish were counted and recorded for all treatments and time points under a high-powered epifluorescence stereoscope (Leica M165-FC, Leica Microsystems, Wetzlar, Germany). Captured images were used to visualize the overall differences in the number of neuromasts counted across each time point. Visible neuromasts were labeled as functional if they appeared to have all or some (>10%) hair cells intact (Fig. 2). Neuromasts with low fluorescent were recorded as “functional” with the assumption that hair cell synapses can still transmit flow stimuli with partial loss (or dysfunction) of the hair cells. Because nonfunctional neuromasts do not fluoresce (Van Trump et al. 2010; Schwalbe et al. 2016), this negative staining is an indication of the effectiveness of each of the chemicals in ablating the neuromasts. The neuromast counts were converted into percentages, and the percentages of superficial and canal neuromasts were compared as time series data for each chemical. We used fluorescence of the olfactory tissue in the nares as a positive control for 4-Di-2-ASP, since it positively stains olfactory tissue, and this tissue is not negatively affected by the dosages of the chemicals used in this study.

### Statistics

We used a log-rank test (MATLAB R2020a, Mathworks, Natick, MA, USA) to compare the differences of the dose-response curves between the time series of each treatment group (streptomycin, neomycin, gentamycin, CoCl_2_, and sham) during observations of ablation and regeneration experiments. Since our sample size is small (*n* = 3 at each time point), we still found value in reporting the means and standard deviations at each time point to show the overall data range and time series trend.

## Results

### The lateral line system of giant danios

Giant danios have a well-defined lateral line system with superficial and canal neuromasts distributed on the head and on the body (Fig. 1, Mekdara et al. 2018). On the dorsal region of the head, giant danios have two large clusters of superficial neuromasts in between the eye orbits and two clusters of superficial neuromasts rostral and lateral to their nares (Fig. 1A and B). Canal neuromasts on the head reside in bony canals that are located anterior (supraorbital canal) and posterior (infraorbital canal) to the eye orbits, on the mandible (mandibular canal), on the gill cover (preopercular canal), on the dorsal region of the head (supratemporal canal), and caudally toward the trunk (optic and postotic canals) (Fig. 1). Like most lateral line systems, canal neuromasts in bony canals and on lateral line scales are located between two canal pores, which allow water to enter the canals. On the body, the trunk canal originates at the dorsal edge of the operculum and extends ventrally along the body from the operculum, posterior to the pectoral fin, and to the base of the caudal fin (Fig. 1). Each trunk lateral line scale has one canal neuromast and several accessory superficial neuromasts above and below the canal. Vertical stitches of superficial neuromasts are located along the length of the body on scales, and there are four to six horizontal lines between the fin rays on each side of the caudal fin (Fig. 1).

### All chemicals ablate the lateral line system

**Fig. 3:**
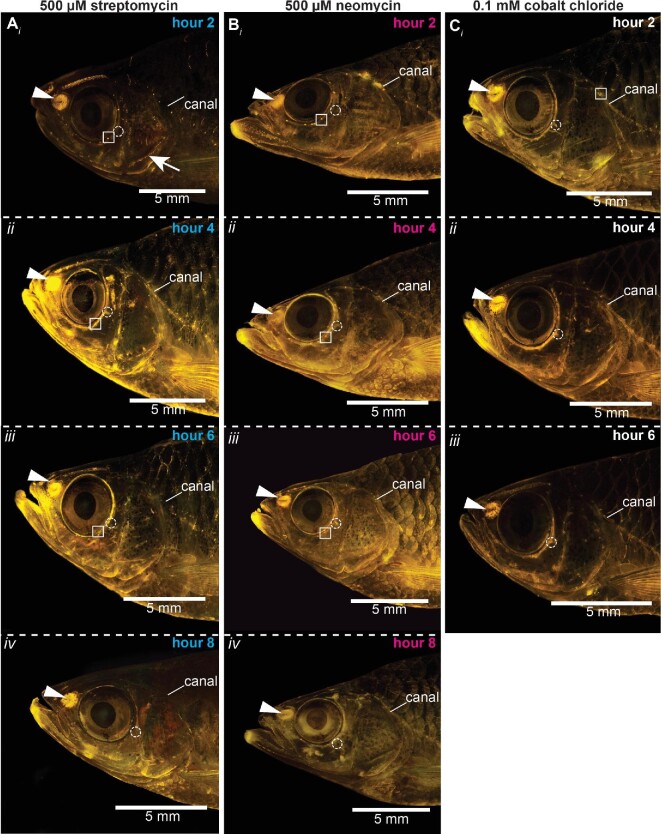
Time course of 500 μM streptomycin, 500 μM neomycin, and 0.1 mM CoCl_2_ treatments through complete ablation (≤8 h). Adult giant danios were treated with one or two concentrations of streptomycin, neomycin, CoCl_2_, or standard tank water (= control and subsets of fish were stained with 4-Di-2-ASP at 2-h intervals until hair cells in neuromasts were completely ablated. Active neuromasts appear as bright yellow dots, while inactive neuromasts do not fluoresce. Olfactory epithelium visible through the nares can also be seen (white triangle notch) because it also stains with 4-Di-2-ASP and serves as a positive control for neuromast staining. Examples of fish treated with (**A**) 500 μM streptomycin at (i) 2, (ii) 4, (iii) 6 , and (iv) 8 h; (**B**) 500 μM neomycin at (i) 2, (ii) 4, (iii) 6, and (iv) 8 h; and (**C**) 0.1 mM CoCl_2_ at (i) 2, (ii) 4, and (iii) 6 h. In the 500 μM streptomycin and neomycin treatments, note overall diminishing fluorescence in superficial neuromasts on the head at 2 h (especially Ai, the white arrow) and full superficial neuromast ablation at 4 h. Canal neuromasts are fully ablated at 8 h. In the 0.1 mM CoCl_2_ treatment, both superficial and canal neuromasts are ablated at 6 h. White squares and white dashed circles track the same canal neuromast and canal pore, respectively, across individuals through sequential images to highlight the loss of staining in these canal neuromasts while the pores remain visible throughout the treatments. Figure 1 shows an example of a control fish.

**Fig. 4:**
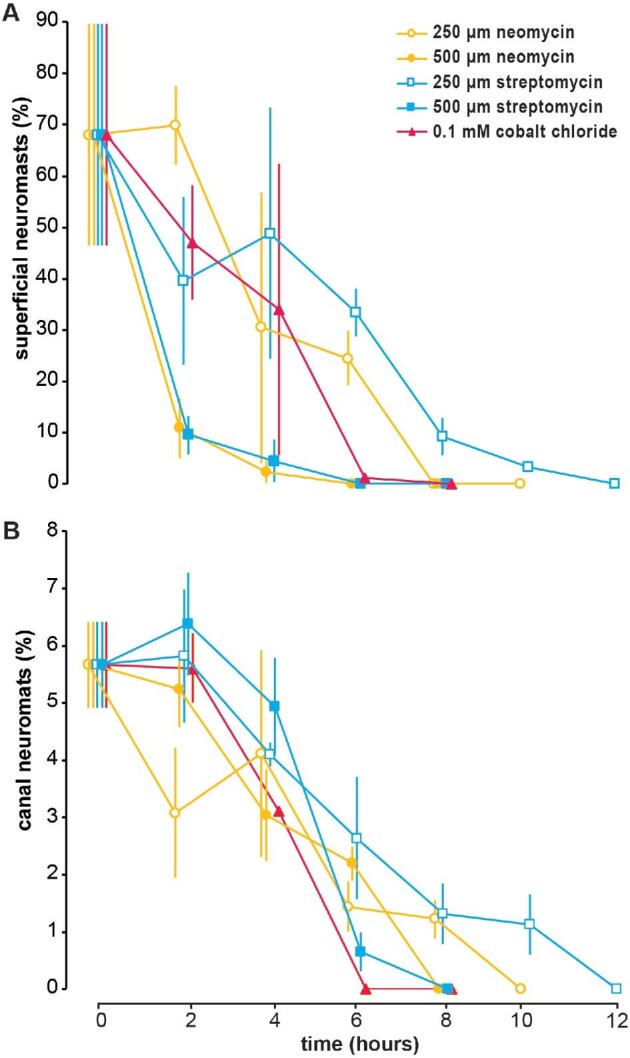
Treatment with 0.1 mM CoCl_2_ ablated hair cells in both superficial and canal neuromasts more rapidly than dosages of both streptomycin and neomycin (250 and 500 μM) in adult giant danios. Positively stained superficial and canal neuromasts were counted on one side of a treated fish (*n* = 3 fish per time point) immediately after treatment (0 h) and every 2 h until completely ablated (≤14 h). The mean percentage of visible (**A**) superficial neuromasts and (**B**) canal neuromasts, both relative to the combined number of neuromasts, decreased over time. The percentages at 0 h do not sum to 100 because of individual variations.

Neomycin, streptomycin, and CoCl_2_ were effective at ablating the lateral line system at all dosages, but with different time courses (Figs. 3 and 4). Ablation was indicated by the lack of fluorescence staining in hair cells (<10% of hair cells in neuromasts; Fig. 2), indicating loss of hair cell function in treated fish. All treatments and time points were significantly different from the sham-treated fish at 0 h ( = control) (*P* < 0.001; *n* = 3 fish per time point). Figure 3 shows examples of positive, partial, and negative fluorescence stained neuromasts on the head at several time points during treatments with 500 μM streptomycin, 500 μM neomycin, and 0.1 mM CoCl_2_. Figure 4 shows the time course of ablation for all treatments. The 250 μM neomycin treatment ablated all neuromasts within 10 h, and the 250 μM streptomycin treatment ablated all neuromasts within 12 h (Fig. 4). Both 500 μM treatments of neomycin and streptomycin ablated all neuromasts within 8 h (Figs. 3 and 4). The 0.01 mM CoCl_2_ treatment ablated all neuromasts within 6 h.

### Treatments with 500 μM neomycin and streptomycin ablate superficial neuromasts faster than canal neuromasts

Superficial neuromasts were ablated earlier than canal neuromasts in the 500 μM neomycin and streptomycin treatments (*P* < 0.001; compare solid and dashed lines in Fig. 4). Superficial neuromasts were ablated and canal neuromasts appeared largely intact (50–80% still fluorescing) after 2–4 h and both neuromasts were fully ablated after 6–8 h (Figs. 3 and  4). In contrast, this trend was not observed in the 250 μM neomycin, 250 μM streptomycin, and 0.1 mM CoCl_2_ treatments. The numbers of fluorescent hair cells in superficial and canal neuromasts declined at approximately the same rate during their respective treatments (Fig. 4), indicating that these dosages affected both types of neuromasts similarly during each treatment.

### The lateral line system regenerates quickly regardless of ablation chemical

**Fig. 5:**
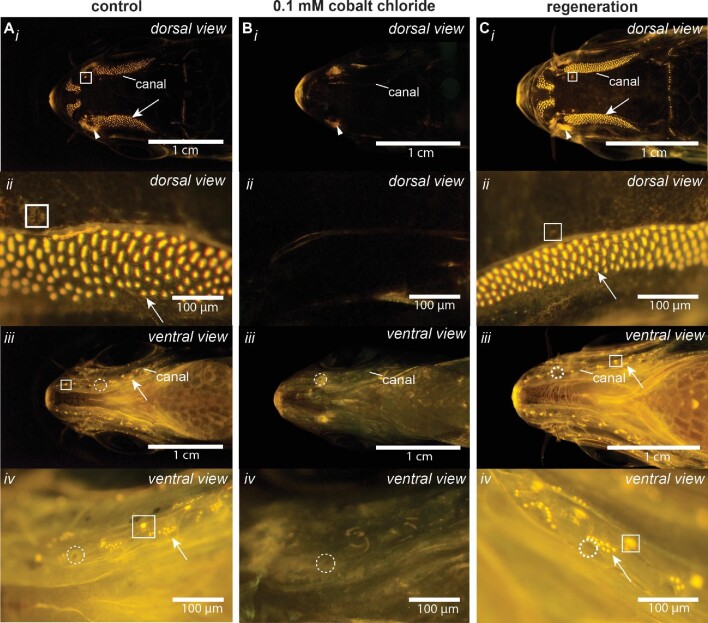
The mechanosensory lateral line system of adult giant danios through ablation with 0.1 mM CoCl_2_ and regeneration. Giant danios were stained with 4-Di-2-ASP, showing functional neuromasts as bright yellow dots. The columns of images show (**A**) sham-treatment fish (= control, after 14 h), (**B**) fish with 0.1 mM CoCl_2_ after 6 h (= complete ablation), and (**C**) fish 7 days post-treatment (= regeneration) with (i–ii) dorsal and (iii–iv) ventral views of the head. Examples of canal neuromasts are in white boxes, superficial neuromasts are indicated with a white arrow, and canal pores are enclosed with a white, dashed circle. Olfactory epithelium visible through the naris can be seen in (Ai)–(Ci) (white triangle notch) because it also stains with 4-Di-2-ASP and serves as a positive control for neuromast staining. Scale bars are 1 cm in dorsal and lateral views and 100 μm where indicated.

**Fig. 6:**
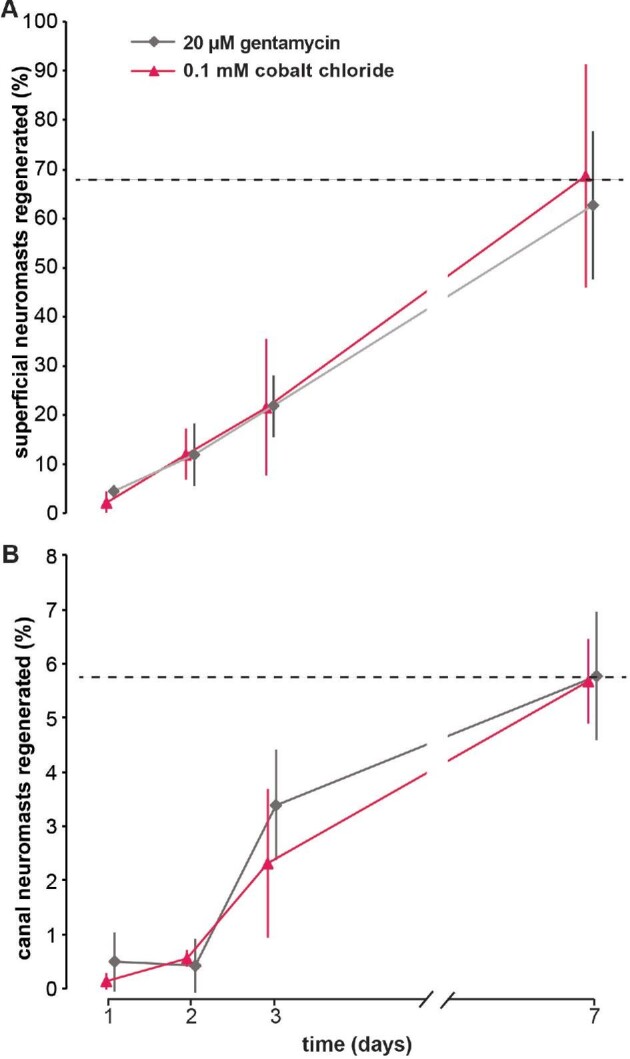
Time course of hair cell regeneration in neuromasts is comparable across 0.1 mM CoCl_2_ and 20 μM gentamycin treatments. Fish were treated with 0.1 mM CoCl_2_ for 4 h or 20 μM gentamycin for 24 h and stained with 4-Di-2-ASP at 1, 2, 3, or 7 days post-treatment (*n* = 3 fish per time point). The mean percentage of visible (>10% hair cells fluorescing) (**A**) superficial neuromasts and (**B**) canal neuromasts, both relative to the combined number of neuromasts, increased over time. The lateral line system of giant danios requires 7 days for complete regeneration of hair cells in neuromasts regardless of ototoxic chemical treatment. The dashed lines indicate the mean percentages of superficial and canal neuromasts before ablation (= control) and do not sum to 100% because of individual variation.

The 0.1 mM CoCl_2_ 4 h treatment and 20 μM gentamycin 24 h treatment ablated all neuromasts (<10% fluorescence). One day after removal from treatment, we recorded some positive fluorescence (>10% fluorescence) (Figs. 5 and 6). The return of positive fluorescent staining indicated hair cell regeneration. Figure 5 compares images from sham-treated fish (control), hair cell ablation in 0.1 mM CoCl_2_ and 20 μM gentamycin treatments, and regeneration of hair cells after 7 days following the treatments. Figure 6 shows the number of neuromasts regenerated after ablation of the lateral line system from 0.1 mM CoCl_2_ and 20 μM gentamycin treatments (*n* = 3 per time point). Only a small number of neuromasts were regenerated at day 1, but by 7 days, all hair cells were fully regenerated (100% fluorescence) following both treatments (Figs. 5 and 6; *P* = 0.524 compared to control).

## Discussion

Several chemicals are frequently used to reversibly ablate the hair cells of the lateral line system in fishes so that its role in various behaviors can be studied. Here, we screened four common ablation agents using giant danios to quantify both the time course to full ablation, in which all of the neuromasts are not functional, and the regeneration of the lateral line after ablation. Aminoglycoside antibiotics (neomycin, streptomycin, and gentamycin) have been used in several studies to ablate neuromasts in larval zebrafish (Song et al. 1995; Harris et al. 2003; Murakami et al. 2003; Owens et al. 2009; Van Trump et al. 2010; Stengel et al. 2017), but few studies have examined the effectiveness of these chemicals in adult fish (but see Song et al. 1995 [streptomycin, gentamycin]; Van Trump et al. 2010 [gentamycin]). Heavy metal ions, such as CoCl_2_ and copper sulfate (CuSO_4_), have also been used regularly to ablate the lateral line system of fish (Karlsen and Sand 1987; Montgomery et al. 1997; Baker and Montgomery 1999; Janssen 2000; Olivari et al. 2008; Schwalbe et al. 2016; Stewart et al. 2017). High concentrations of CoCl_2_ >0.1 mM can be toxic to fish and cause other tissue damage that may negatively impact behavioral studies (Janssen 2000; Butler et al. 2016). When selecting a chemical for lateral line ablation, researchers should select the best chemical for their experiments based on concentration and exposure time to minimize any potential harm and stress for their study species.

We found the following: (1) All chemicals were effective at ablating lateral line hair cells in giant danios. The 0.01 mM CoCl_2_ treatment caused the most rapid ablation, and it ablated all hair cells in 6 h, while the aminoglycoside antibiotic treatments took 8–12 h depending on dosage. (2) Dosages of 500 μM neomycin and streptomycin can ablate superficial neuromasts in 2 h and leave canal neuromasts mostly intact if chemical treatments stop at 2 h. (3) Complete regeneration of the neuromasts requires 7 days after ablation, regardless of the ablation chemical used.

### Aminoglycoside antibiotics ablate all hair cells in neuromasts but can ablate superficial and canal neuromasts at different rates

Hair cells of the lateral line system in fish are ablated by aminoglycoside antibiotics. These chemicals can disrupt hair cell signaling by either blocking the mechanotransduction channels (Kroese and van den Bercken 1982; Ernst et al. 1994; Forge and Schacht 2000) or through degeneration of apical cilia, both of which trigger processes leading to hair cell death via apoptosis (Williams et al. 1987; Forge and Schacht 2000). We found that both streptomycin and neomycin can fully ablate all of the hair cells in the lateral line system at a similar rate like gentamycin (Van Trump et al. 2010).

However, at a relatively high concentration, both neomycin and streptomycin (500 μM) ablated hair cells in superficial neuromasts faster than those in canal neuromasts (Fig. 4). These high concentrations can ablate hair cells in superficial neuromasts within 2–4 h, while hair cells in canal neuromasts are mostly intact. This suggests that hair cells within the two types of neuromasts may have different degrees of sensitivity to aminoglycoside antibiotics. In previous studies, gentamycin was thought to have the opposite effect, ablating hair cells in canal neuromasts but not superficial (Song et al. 1995), but a later study showed that it equally affects hair cells in both types of neuromasts (Van Trump et al. 2010).

Another explanation involves how the hair cells are exposed to the ablation chemical. Since canal neuromasts are located in pored canals, it may simply take longer for the chemical to reach the neuromast through the small canal pores, narrow canals, and body mucus. Thus, longer exposures, regardless of concentration, may be necessary to ablate all hair cells in canal neuromasts, while at high dosage (500 μM), the hair cells in superficial neuromasts are ablated more rapidly. Our work suggests that hair cells in superficial neuromasts can be nearly entirely ablated while leaving hair cells in canal neuromasts mostly intact, but one should proceed with caution if using this ablation strategy. Even at relatively short treatment exposures (e.g., <4 h), the aminoglycoside antibiotics do ablate hair cells in both types of neuromasts to some degree, and the amount of hair cell ablation may vary across individuals (Figs. 3 and 4).

### Using CoCl_2_ to ablate the lateral line system

As in previous studies (Montgomery et al. 1997; Baker and Montgomery 1999; Stewart et al. 2013; Schwalbe and Webb 2014; Mekdara et al. 2018), our fluorescent data show that 0.1 mM CoCl_2_ ablates the lateral line system but leaves the olfactory epithelium in the nares intact (as indicated by positive staining at 6 h, Fig. 3C iii). The difference between our results and those of Butler et al. (2016) supports the hypothesis that there is variation among species on the effects of CoCl_2_ on olfactory epithelium and chemosensory cells (Janssen 2000; Butler et al. 2016). Prior to behavioral studies with a new fish species, we encourage preliminary tests to evaluate how CoCl_2_ may impact these additional sensory systems.

### Regeneration of the neuromasts

The hair cells in neuromasts of the lateral line system can regenerate after ablation, a process well documented in zebrafish (reviewed in Monroe et al. 2015) and other teleosts (Becker 2013; Schwalbe et al. 2016), but it was unclear if hair cells regenerate at different rates depending on the ablation chemical. After ablation with either aminoglycoside antibiotics or a heavy metal ion, hair cells can rapidly regenerate through proliferation and cell division or by the conversion of support cells into hair cells (Balak et al. 1990; Harris et al. 2003; Hernández et al. 2007). Ablation using a heavy metal ion could potentially result in slower regeneration than ablation using an aminoglycoside antibiotic. This is because the heavy metal ion tends to ablate the support cells as well, and without the support cells, regeneration may take longer than when they remain intact (Balak et al. 1990; Hernández et al. 2007; Thomas et al. 2015). We examined and compared hair cell regeneration following treatments of aminoglycoside antibiotics and CoCl_2_, a heavy metal ion in solution, and found no difference between treatments: the rate of regeneration of hair cells in neuromasts was similar between both chemicals. Regeneration from either treatment resulted in a similar time course toward full recovery of hair cells in both superficial and canal neuromasts (Fig. 6).

## Conclusion

Our results continue to support that several aminoglycoside antibiotics (neomycin, streptomycin, and gentamycin) and a heavy metal (CoCl_2_) effectively ablate hair cells in superficial and canal neuromasts of the lateral line system. Further, the time course of hair cell regeneration was comparable between these treatment types. These chemicals are accessible and relatively inexpensive and are safe to use on a variety of fish species at dosages appropriate for their life stage. Care should be taken when selecting which chemical to use to reversibly ablate the lateral line system as there are trade-offs between these chemicals. CoCl_2_ fully ablates hair cells in both superficial and canal neuromasts relatively quickly (≤6 h) but may impair olfactory tissue and chemosensory cells. Aminoglycoside antibiotics take longer to fully ablate hair cells in both types of neuromasts (≤12 h), and lower concentrations (e.g., 250 μM) take longer than higher concentrations (e.g., 500 μM). These chemicals do not appear to negatively impact other sensory systems. Higher dosages of aminoglycoside antibiotics (500 μM) may ablate hair cells in superficial neuromasts before those in canal neuromasts, but true selective ablation of one type of neuromast while the other is entirely intact is unlikely to be achieved. While this study focused on ablation treatments commonly used in behavioral studies in fish, it can also contribute to hearing research involving ototoxic chemicals and their effects on hair cells in vertebrate acoustic and vestibular systems.
